# The influence of secondary structure, selection and recombination on r*ubella virus* nucleotide substitution rate estimates

**DOI:** 10.1186/1743-422X-11-166

**Published:** 2014-09-16

**Authors:** Leendert J Cloete, Emil P Tanov, Brejnev M Muhire, Darren P Martin, Gordon W Harkins

**Affiliations:** South African National Bioinformatics Institute, SA Medical Research Council Unit for Bioinformatics Capacity Development, University of the Western Cape, Cape Town, South Africa; Institute of Infectious Diseases and Molecular Medicine, Computational Biology Group, University of Cape Town, Cape Town, South Africa

**Keywords:** Rubella virus, Congenital rubella syndrome, Nucleotide substitution rates, Synonymous substitution rates, Recombination, Nucleic acid secondary structure, Bayesian phylogenetic analyses

## Abstract

**Background:**

Annually, rubella virus (RV) still causes severe congenital defects in around 100 000 children globally. An attempt to eradicate RV is currently underway and analytical tools to monitor the global decline of the last remaining RV lineages will be useful for assessing the effectiveness of this endeavour. RV evolves rapidly enough that much of this information might be inferable from RV genomic sequence data.

**Methods:**

Using BEASTv1.8.0, we analysed publically available RV sequence data to estimate genome-wide and gene-specific nucleotide substitution rates to test whether current estimates of RV substitution rates are representative of the entire RV genome. We specifically accounted for possible confounders of nucleotide substitution rate estimates, such as temporally biased sampling, sporadic recombination, and natural selection favouring either increased or decreased genetic diversity (estimated by the PARRIS and FUBAR methods), at nucleotide sites within the genomic secondary structures (predicted by the NASP method).

**Results:**

We determine that RV nucleotide substitution rates range from 1.19 × 10^-3^ substitutions/site/year in the E1 region to 7.52 × 10^-4^ substitutions/site/year in the P150 region. We find that differences between substitution rate estimates in different RV genome regions are largely attributable to temporal sampling biases such that datasets containing higher proportions of recently sampled sequences, will tend to have inflated estimates of mean substitution rates. Although there exists little evidence of positive selection or natural genetic recombination in RV, we show that RV genomes possess pervasive biologically functional nucleic acid secondary structure and that purifying selection acting to maintain this structure contributes substantially to variations in estimated nucleotide substitution rates across RV genomes.

**Conclusion:**

Both temporal sampling biases and purifying selection favouring the conservation of RV nucleic acid secondary structures have an appreciable impact on substitution rate estimates but do not preclude the use of RV sequence data to date ancestral sequences. The combination of uniformly high substitution rates across the RV genome and strong temporal structure within the available sequence data, suggests that such data should be suitable for tracking the demographic, epidemiological and movement dynamics of this virus during eradication attempts.

**Electronic supplementary material:**

The online version of this article (doi:10.1186/1743-422X-11-166) contains supplementary material, which is available to authorized users.

## Background

Rubella virus (RV), the sole species in the genus *Rubivirus* of the family *Togaviridae*, is the causative agent of a highly contagious airborne disease that is most commonly known in the western world as either rubella or German measles. Despite RV having been virtually eliminated in many countries [1–3], CRS and childhood rubella are endemic across much of South-East Asia and Africa with over 100 000 cases of CRS estimated to occur around the world annually. In response to the devastating human and socio-economic costs of this disease, the World Health Organization (WHO) is aiming for the complete eradication of RV by 2020 [4].

The urgent need for effective rubella vaccination programs was underscored by the global pandemic in 1962 [5] and the first of these programs was initiated in the USA in 1969-70. By 2010, 68% of the WHO Member States included rubella vaccines in their routine immunization programs [4]. Because of the uneven adoption and coverage of rubella control programs among countries around the world, RV infections constitute a significant on-going global health threat.

RV is an enveloped virus with a positive-sense, single-stranded RNA genome ~ 9,762 nucleotides in length. Its genome has two open reading frames (ORFs) with the 5’ ORF encoding the non-structural proteins (NSP; P150 and P90) that function in RNA replication, and the 3’ ORF encoding the structural proteins (SP; capsid protein, CP, and two envelope glycoproteins, E1 and E2) that together make up the virion (see Additional file 1). RV is also unique in the fact that its genome has the highest genomic GC content (~70%) of all known RNA viruses [6].

Two major clades of RV exist with constituent members differing from one another at between 8 and 10% of genomic sites. Whereas clade 1 consists of nine recognised and one provisional (designated by lower case letter) RV genotypes (1a, 1B, 1C, 1D, 1E, 1 F, 1G, 1H, 1I, and 1 J), clade 2 contains three recognised genotypes (2A, 2B and 2C) [7–9]. Clade 2 genotypes were presumably restricted to Asia until the 2000s [10]. However, genotype 2B viruses have subsequently become widely distributed geographically, and together with 1E and 1G, are the genotypes most frequently found among the more recent samples [11].

Besides increased volumes of genomic sequence data, an important prerequisite for using RV sequences in such surveillance efforts is the demonstration that the rates at which RV genomes are evolving are both high- and constant enough, that they can be reliably used to date both epidemiologically relevant fluctuations in virus population sizes, and viral movement events (such as transmissions between individuals or migrations between different countries or continents). In this regard, it is very promising that RV E1 encoding genome region sequences display high degrees of clock-like evolution and mean nucleotide substitution rates ranging between 6.1 × 10^-4^ [12] and 1.65 × 10^-3^ substitutions per nucleotide site per year [13] - a rate of evolution that should be within the bounds required to extract meaningful phylogeographic and demographic information from RV genomic sequence data. It is noteworthy, however, that whereas nucleotide substitution rates that have been estimated for other togaviruses using the same strict-clock maximum likelihood-based methods employed for the RV E1 encoding region [12], are substantially lower than those estimated for RV, other studies [13] using more sophisticated Bayesian relaxed molecular clock–based inference methods have reported that the RV E1 substitution rate is approximately equivalent to those of other togaviruses [14–16].

Using publically available RV gene and full genome sequences sampled over the past 51 years we here attempted to test whether current estimates of RV substitution rates are representative of the entire RV genome. During these investigations we specifically accounted for possible confounders of nucleotide substitution rate estimates such as temporal sampling biases, sporadic genetic recombination and natural selection favouring either increased genetic diversity in response to host immune pressures, or decreased genetic diversity at nucleotide sites involved in the formation of nucleic acid secondary structures.

## Results and discussion

### Identification of nucleic acid secondary structures within RV genomes

Nucleic acid secondary structures are created through the formation of hydrogen bonds between complementary bases of the nucleotide sequence. Extensive nucleic acid secondary structure exists within the genomes of many mammalian and plant single-stranded RNA viruses [17] with the most biologically relevant structural elements within these molecules being highly conserved.

**Figure 1:**
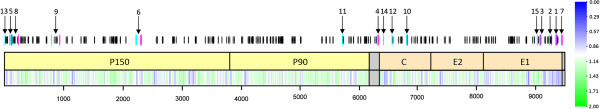
**Genome-wide predicted high confident structure set and synonymous substitution rates.** Pairs of vertical lines above the genome represent the stem regions of the high confidence structure set (HCSS; Additional file 2). The cyan and magenta vertical lines indicate the fifteen highest consensus ranked structures (as estimated using the highest average ranking across all consensus ranking tests performed, see Methods), with the cyan line representing the 5’-most stem of the structure – and the magenta the 3’ (single cyan lines represent structures to small to be displayed in their entirety at the current image scale). Nucleotide positions are shown on the x-axis. The vertical lines below the gene map indicate site-to-site variation in synonymous substitution rates (see colour key). Blue and green coloured lines represent codon sites having lower and higher than expected (elevated or reduced rate, relative to the mean) synonymous substitution, respectively.

Selection favouring the maintenance of nucleic acid secondary structural elements could potentially influence our substitution rate estimates. In order to account for these potentially confounding effects, we used the computer program NASP v1.5 [18, 19] to identify evolutionarily conserved base-paired sites within ten full length RV genomes sampled from each of the most representative RV lineages (dataset i, see Methods section. Overall mean genetic distance between lineages: 6.9%). NASP identified 661 potentially conserved nucleic acid secondary structural elements; 121 of them, account for >95% of the difference in thermostability between the observed sequences and the randomised versions of the sequences. Collectively these formed the high confidence structure set (HCSS) upon which we focused further analyses. Approximately 21% of the nucleotides within the 121 conserved structural elements of the HCSS are likely base-paired (Figure 1, Additional files 2 and 3).

Well-supported nucleic acid secondary structural elements within the HCSS were identified in both the NSP and SP ORFs with the majority occurring in the SP ORF. All four of the previously characterised RV genomic structural elements (within RV coding regions) were within the top 20 of those highlighted in the DOOSS consensus ranking. In this ranking, structures are ordered according to their associated degrees of conservation, synonymous substitution rate reduction at codon sites containing paired nucleotides and the amount of evidence for complementary coevolution between nucleotides predicted to be base-paired (see Methods section). In the SP coding region two well-characterized structural elements known to be involved in calreticulin binding [20] were ranked first and seventh. Similarly, the structural element serving as a template for the sub-genomic RNA promoter on the negative-sense strand was ranked fourth [21]. Another structural element straddling the 5’UTR and the NSP P150 coding region, that promotes genomic positive strand synthesis [22], was ranked eighteenth. Notably, whereas four of the 10 top ranked structures were situated within the E1 gene region (including the three highest ranked structures), none of the top 20 ranked structures were located in the E2 non-structural glycoprotein region.

### Synonymous substitution rate- and nucleotide coevolution selection tests at paired- vs. unpaired sites

However, given the very high GC contents of RV genomes, it is expected that they will have a reasonably high degree of nucleic acid secondary structure irrespective of any potential roles on the biology of this virus. If most of the detected structural elements have no biological function, then there should be little evidence of natural selection operating to maintain these structures. If, however, base-paired nucleotides within structural elements are either evolving under stronger negative selection than unpaired sites (selection against substitutions, i.e. they are evolving “less-neutrally”), or are co-evolving with their pairing partners (i.e. they are evolving non-independently), this could plausibly have an effect on nucleotide substitution rate estimates.

To test this hypothesis we used the FUBAR [23] and PARRIS [24] methods to estimate synonymous substitution rates within the RV NSP and SP coding regions (see Figure 1). We specifically tested for evidence of selection against synonymous substitutions at codons containing paired nucleotides at their third positions (referred to as “paired codon sites”). Using a Mann-Whitney U-test, we compared median estimated substitution rates at paired and unpaired codon sites. These tests revealed that both the SP and NSP coding regions displayed significantly lower nucleotide substitution rates at paired codon sites than at unpaired codon sites (PARRIS p-value = 2.288 × 10^-2^ and FUBAR p-value = 4.068 × 10^-5^ for the SP and PARRIS p-value = 5.205 × 10^-3^ and FUBAR p-value = 1.118 × 10^-6^ for the NSP).

To further test whether base-paired sites were co-evolving so as to maintain base-pairing complementarity, we used a SPIDERMONKEY-based method. This method identifies co-evolving nucleotide pairs, which act to maintain complementary base-pairings. We found a significant association between NASP predicted base-paired sites within the HCSS and genomic sites predicted by the SPIDERMONKEY-based method [25, 26] to be coevolving with one another in a complementary fashion (p = 2.2 × 10^-16^). Although this finding suggests that a large proportion of nucleotides within RV genomes are not independently evolving, it is not possible to quantify the ratio of sites co-evolving against those that are not, using this method.

These results show that there are 116 previously unreported structures, predicted by NASP, within the RV coding regions that are likely biologically relevant and that their constituent nucleotides are not evolving in a strictly neutral fashion. It is however not possible to determine, based on these analyses, which individual structural elements are functional.

### Recombination within RV genome sequences

Since recombination undermines the accuracy of phylogenetic inference [27, 28], and some evidence of recombination has previously been reported in RV sequences that are deposited in sequence databases [29–31], we opted to scan our datasets for evidence of recombinant sequences. Collectively, we detected evidence of only two recombinant sequences (GenBank:JN635285 [31] and GenBank:AF435866 [32]).

**Figure 2:**
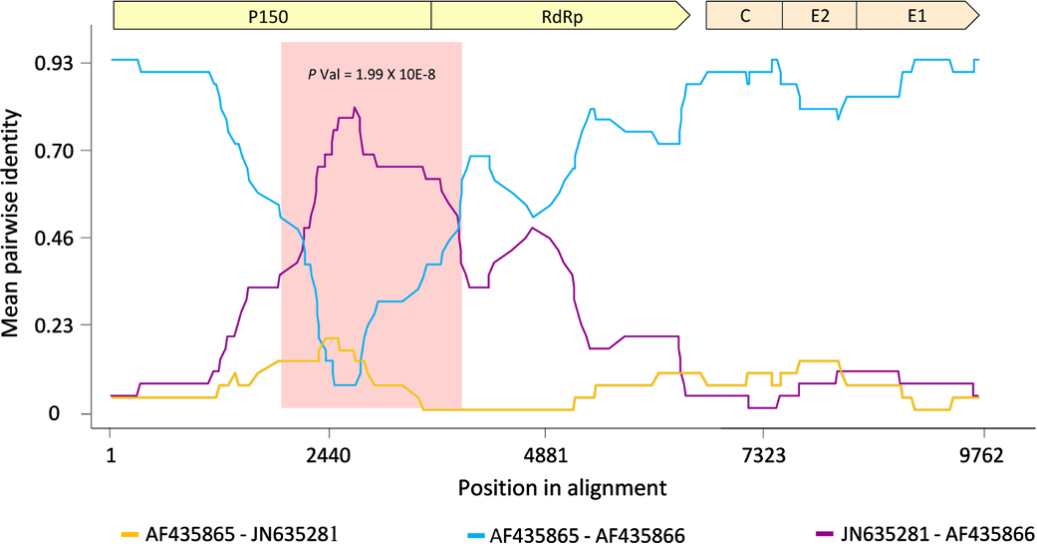
**Pairwise identity plot of the potential recombination event detected in the full genome RV dataset.** The non-structural and structural coding regions are shown above the plot, in blue and green respectively (plot scale drawn with respect to isolate [GenBank:AF435866]). The y-axis represents the mean pairwise identity between the sequences within a 30-nucleotide window moved one nucleotide at a time along the length of the genome. Pairwise comparisons between the major [GenBank:AF435865; isolate contributing a larger segment of nucleotide sequence] and minor [GenBank:JN635281; isolate contributing a smaller segment of nucleotide sequence] parents are shown in orange, between the major parent and recombinant [GenBank:AF435866] in cyan, and between the minor parent and recombinant sequences, in purple. The area outlined in pink demarcates the potential recombinant region (*P* value < 0.05).

We detected significant evidence for an inter-genotype recombination event with breakpoint positions at approximately nucleotides 715 to 2768 located within the NSP P150 gene region of sequence [GenBank:JN635285], which is inconsistent with the results of Abernathy et al. [31]. We also detected a previously unreported intra-genotype (1a) recombination event involving approximate breakpoint positions at nucleotide sites 2017 and 4219 within the P150 NSP region of [GenBank:AF435866] (Figure 2). This genome is currently provisionally classified as a genotype 1a sequence and has not been previously investigated for evidence of recombination using full genome RV sequence data. It is noteworthy that the sequences of [GenBank:AF435866], and the isolate amongst all those analysed which was identified by RDP4.17 [33] as being most closely related to its parent, [GenBank:AF435865], were both determined in the same laboratory [32] – a fact which suggests that [GenBank:AF435866] may be a laboratory artefact rather than a genuine natural recombinant [34]. A further previously unreported recombination event was detected in the E1 region with a single breakpoint located at nucleotide position 8612 nt of [GenBank:AY280706].

### Positive selection within the RV coding regions

In contrast to the results of some previous studies [32], our analysis of selection pressures acting on individual codon sites using the FUBAR method found no significant evidence (highest posterior probability = 0.77 that dN/dS >1) of sites within the RV coding regions that were detectably evolving under positive selection. Instead around 91% of the NSP codon sites and 81% of the SP codon sites were inferred to be evolving under negative selection with posterior probability values of > 0.9: A finding consistent with previous studies [30].

### Temporal structure of RV genome sequences

The degree of clock-like evolution evident within the various sequence datasets was analysed using root-to-tip genetic distance versus sampling date regression analyses with the computer program, Path-O-Gen v1.4 [35, 36]. This revealed high degrees of temporal structure in all datasets as evidenced by correlation coefficients ranging between 0.9 (for the full genome dataset) and 0.67 (for the E1 dataset) [datasets ii and viii, respectively, see Methods section]. In the absence of pervasive recombination and positive selection, this indicated that all of the assembled datasets could be productively used to estimate nucleotide substitution rates and times to the most recent common ancestor (TMRCA’s).

### Nucleotide substitution rates across the RV genome

Also consistent with previous studies [30, 31], the best-fit nucleotide substitution models for the different RV datasets was TN93 with either a calculated proportion of invariant sites (I) or gamma distribution (G). For all analysed datasets (see Additional file 4) the uncorrelated lognormal relaxed-clock models had significantly higher likelihoods than the strict-clock models under both demographic models tested (constant population size, Bayesian skyline plot). However, both demographic models fitted the data equally well.

**Figure 3:**
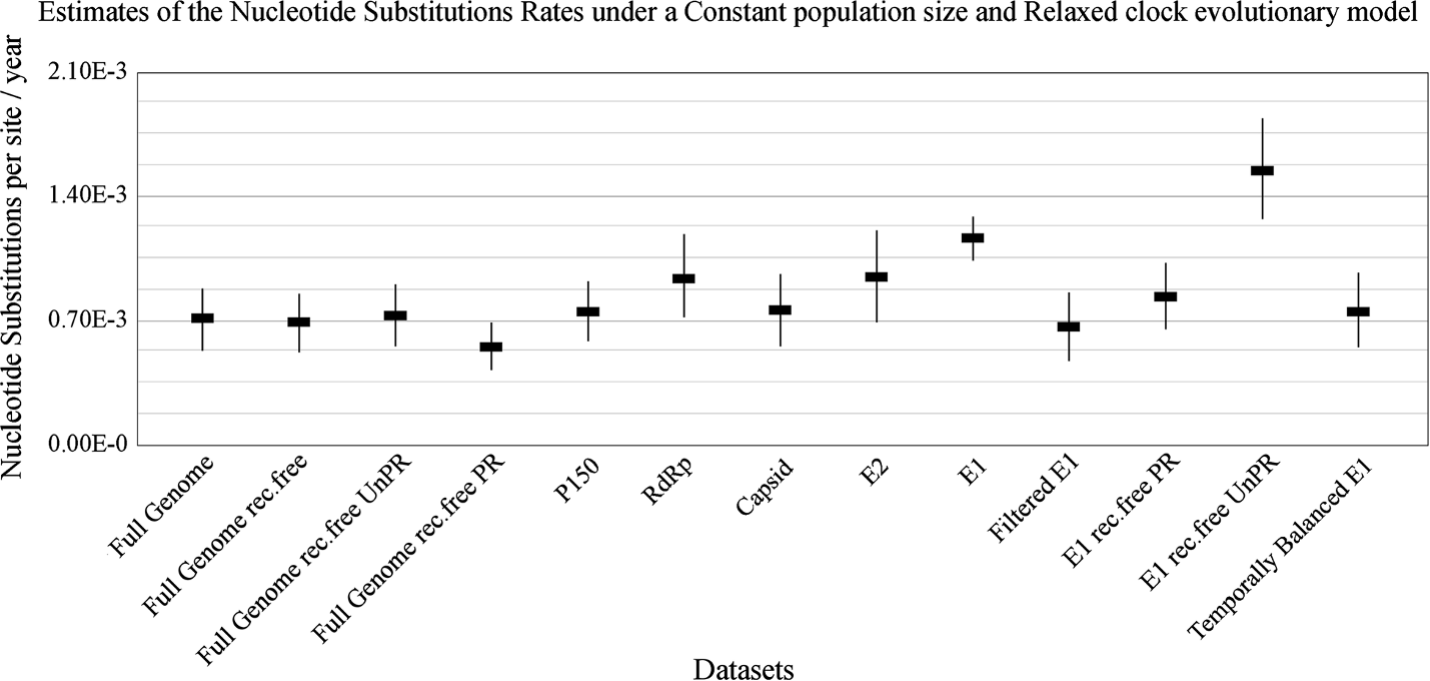
**Nucleotide substitution rate estimates for the different rubella virus sequence datasets.** Nucleotide substitution rate estimates for the different rubella virus datasets under the appropriate nucleotide substitution model run under a constant population size and relaxed-clock evolutionary model.

Of the genomic regions analysed, the E1 structural protein-coding region (1.19 × 10^-3^ substitutions/site/year; 95% HPD = 1.04 × 10^-3^ – 1.35 × 10^-3^) displayed the highest estimated nucleotide substitution rate, and the P150 non-structural protein region the lowest (7.52 × 10^-4^ substitutions/site/year; 95% HPD = 5.85 × 10^-4^ – 9.26 × 10^-4^; Figure 3). All of these estimates, with the exception of the E1 gene (dataset viii, see Methods section), had substantially overlapping 95% HPD’s with the rates reported previously for RV by Jenkins et al. [12]. The E1 gene substitution rate estimate was roughly twice as high as that previously estimated using a dataset of 50 sequences sampled between 1961 and 2001 [12]. All of our estimates were however substantially lower than the rates reported for the E1 gene within the 1E genotype sampled in China between 2001 and 2009 [13].

Similar genome-wide nucleotide substitution rate estimates to those reported here have also been reported for *Chikungunya virus*, another Togavirus in the genus Alphavirus, using the same approach as that used here [14–16]. However, it is impossible to enumerate the proportion of the nucleotide changes represented in our datasets that are transient mutations that will ultimately be purged from the population by genetic drift (or weak purifying selection). It is likely that, due to the inclusion of larger numbers of recently sampled E1 gene sequences than in [12] (only 5% of the 640 samples considered here were collected prior to 1990), our nucleotide substitution rate estimates for this gene are inflated and reflect a composite of the RV basal mutation rate (i.e. the rate at which all mutations occur) and its substitution rate (i.e. the rate at which only persistent mutations occur) [37].

To test this hypothesis we analysed an E1 dataset including only the 34 sequences contained within the full genome sequence dataset [dataset ix, see Methods section]. We found that estimated substitution rates did indeed decrease to become similar to the rates inferred for the other RV genomic regions (see “Filtered E1” in Figure 3). Similarly low substitution rates were also estimated when we analysed a “temporally balanced” E1 dataset [dataset x, see Methods section] containing only a random subset of 45 E1 sequences sampled between 1961 and 2012 (see “Temporally Balanced E1” in Figure 3). These results therefore strongly suggest that substitution rates are not actually higher in E1 than they are in the remainder of the genome.

### Estimated dates of the time to the most recent common ancestor of RV

**Figure 4:**
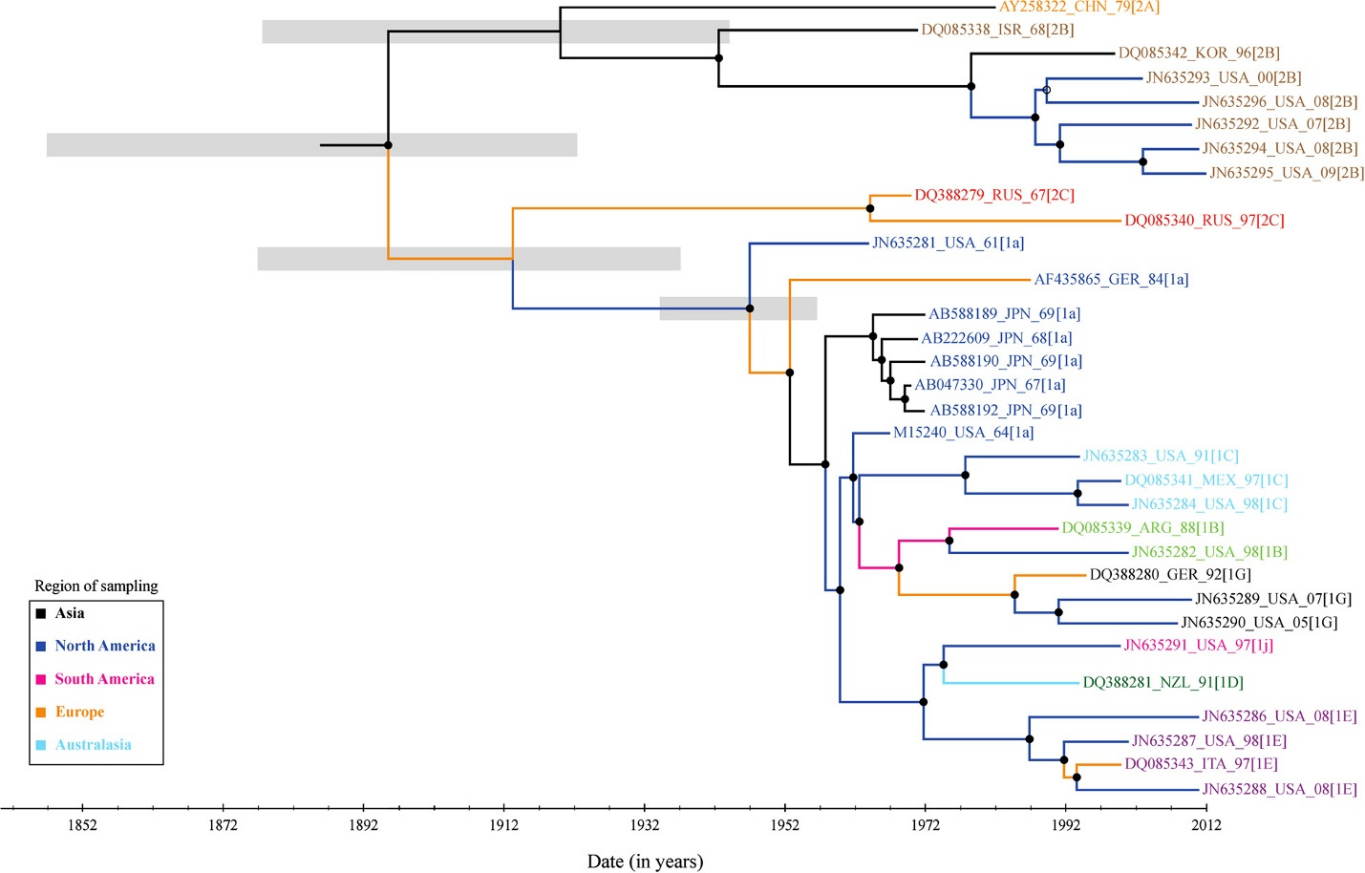
**Maximum clade credibility tree of the full genome recombination-free dataset.** Maximum clade credibility tree constructed from the 32 full recombination-free RV genomes under the TN93 + G + I nucleotide substitution model and the Bayesian skyline plot, relaxed-clock evolutionary model. Branches are coloured according to the region of sampling and the taxon labels according to the genotype. Internal nodes with posterior support greater than 90% are indicated by a filled circle and greater than 80% by an open circle. Thick grey lines at the root and most basal nodes of clade 1 and 2 genotypes, respectively, represent the 95% HPD of the time to the most recent common ancestor.

Regardless of differences between the datasets with respect to estimated substitution rates, the associated estimates of the mean date of the most recent common ancestor for the different RV lineages analysed here all ranged between 1884 (95% HPD = 1841 – 1921) with the full genome dataset and 1926 (95% HPD = 1904 – 1947) with the RdRp dataset (see Figure 4 and Additional file 5). The mean TMRCA estimates for the E1 dataset with the various evolutionary models tested were well within this range (between 1901 and 1911) implying that sampling biases such as those evident in the E1 dataset need not have a particularly large impact on TMRCA estimates.

Also irrespective of the evolutionary model and dataset used, the estimated time to the most recent common ancestor of the clade 2 genotypes was older than that of the clade 1 genotypes. This is consistent with previous reports [10] indicating that, among the sampled sequences, the MRCA of the clade 2 genotypes may have an Asian origin. Finally, it is important to stress that these estimates do not indicate the date when RV first emerged. They simply indicate when the most recent common ancestor of the RV genotypes analysed likely existed.

### The effects of recombination, selection and nucleic acid secondary structure on RV substitution rate estimates

To evaluate the potentially confounding effects of recombination and secondary structure on our estimates of nucleotide substitution rates, we repeated all the substitution rate analyses on the full genome and E1 datasets (dataset ii and viii, respectively), by removing the detected recombinants and all sites that were inferred (within the HCSS) to be involved in base-pairing within secondary structures.

The mean nucleotide substitution rate estimates for the full genome rec.free dataset was similar to the rate inferred from the full genome dataset (Figure 3). Also, when sites inferred to be base-paired within secondary structural elements were removed from the full genome rec.free dataset, the mean substitution rate estimate was not substantially different to the estimates obtained with the full genome rec.free and full genome datasets (compare “Full Genome”, “Full Genome rec.free” and “Full Genome rec.free UnPR”). However, when only the sites inferred to be base-paired were considered, a substantially lower substitution rate was estimated than those estimated with the full genome rec.free datasets (compare “Full Genome rec.free UnPR” and “Full Genome rec.free PR”). Similar results were obtained when the unpaired and paired sites were separately considered in the E1 dataset (compare “E1 rec.free PR” and “E1 rec.free UnPR”) suggesting that the constraints imposed by the combined effects of recombination and nucleic acid secondary structure act to significantly reduce both genome-wide and E1 glycoprotein gene derived nucleotide substitution rate estimates.

## Conclusion

Consistent with the results of previous studies, we have shown that nucleotide substitution saturation has not occurred in RV [30] and that evidence for recombination [29–31] and positive selection [32] is sparse. Despite the fact that the constituent nucleotides in RV genomes are likely not evolving in a strictly neutral fashion, the nucleotide substitution rates estimated here should be sufficiently high that RV sequences sampled worldwide will contain epidemiologically relevant information that should enable the tracking of both population size fluctuations and virus movement dynamics. Although we have demonstrated that temporally biased sampling in RV genome regions such as that encoding the E1 glycoprotein, result in higher mean substitution rate estimates, such biases should have a negligibly negative impact on the utility of E1 sequences for dating ancestral RV sequences under relaxed-clock evolutionary models. This implies that in addition to epidemiological surveillance, RV E1 datasets (representing what is currently the most frequently sampled RV genome region) should contain sufficient phylogenetic signal to be appropriate for sequence-based inferences of RV demographic and movement dynamics.

## Methods

Alignment of all of the RV datasets described below (see Table 1 and Additional file 4) was performed using MUSCLE [38]. Alignments were manually edited using MEGAv5.05 [39]. Fourteen RV multiple sequence alignments were analysed: (i) a full genome dataset, containing a representative sample (10 of the 34 publically available full genome sequences) of RV lineages, was created to predict plausible genome-wide nucleic acid secondary structural elements. These ten sequences were identified using pairwise genetic distances (calculated using SDT v1.0 [40]) and selected from distinct clades within a Neighbour Joining phylogenetic tree (calculated using MEGA v5.05). Only ten of the 34 available full genome sequences were selected for nucleic acid secondary structure analysis to reduce the computational burden imposed by NASP.

**Table 1:** **Summary description of the various datasets used in the study**

Dataset	Description	Acronym	Number of sequences	Temporal range	Alignment length
i	Full genome, representative sample containing 10 rubella virus lineages (extracted from dataset ii)	-	10	1961 - 2008	9762 nt
ii	Full genome (not tested for recombination)	*Full Genome*	34	1961 - 2009	9762 nt
iii	Full genome (without 2 detected recombinant isolates)	*Full Genome rec.free*	32	1961 - 2009	9762 nt
iv	Capsid structural protein	*CP*	52	1961 - 2009	900 nt
v	RNA-dependent RNA polymerase	*RdRp*	56	1961 - 2009	672 nt
vi	Envelope structural glycoprotein 2	*E2*	54	1961 - 2009	846 nt
vii	P150 non-structural protein	*P150*	34	1961 - 2009	3943 nt
viii	Envelope glycoprotein 1	*E1*	640	1961 - 2012	739 nt
ix	*Filtered* envelope glycoprotein 1, extracted from dataset ii	*Filtered E1*	34	1961 - 2009	739 nt
x	Temporally balanced envelope glycoprotein 1	*Temporally Balanced E1*	45	1961 - 2012	739 nt
xi	Envelope glycoprotein 1, without 2 detected recombinant isolates and 437 nt NASP predicted base-paired nucleotide sites	*E1 rec.free UnPR*	638	1961 - 2012	302 nt
xii	Envelope glycoprotein 1, without 2 detected recombinant isolates, containing only 437 nt NASP predicted base-paired nucleotide sites	*E1 rec.free PR*	638	1961 - 2012	437 nt
xiii	Full genome, without 2 detected recombinant isolates and 1960 nt NASP predicted base-paired nucleotide sites.	*Full Genome rec.free UnPR*	32	1961 - 2009	7802 nt
xiv	Full genome, without 2 detected recombinant isolates, containing only 1960 nt NASP predicted base-paired nucleotide sites	*Full Genome rec.free PR*	32	1961 - 2009	1960 nt

For genome-wide nucleotide substitution rate estimates, we created (ii) a full genome dataset containing 34 full genome sequences and (iii) a full genome mostly “recombination-free” (rec.free) dataset containing 32 full genome sequences from which sequences identified as having been derived through recombination using the computer program RDP4.17 [33] were excluded. At the time of the analysis, the 34 sequences were the only available full genome sequences on GenBank, excluding the vaccine strains and multiple sequences from certain isolates. Since we aimed to test the effect of recombination on the estimation of the RV nucleotide substitution rates, we opted to create both full genome datasets either containing or excluding sequences identified as having been derived through recombination, respectively.

For the NSP and SP datasets, the various genes were excised from the 34 full genome sequences, and supplemented by additional sequences from GenBank for the specific gene of interest, if any were available. The result being (iv) a Capsid gene dataset (CP) containing 52 CP encoding sequences (v) a 672 nt RNA-dependent RNA polymerase (RdRp) dataset containing 56 sequences (vi) a E2 gene dataset (E2) containing 54 sequences (vii) a P150 gene dataset containing 34 sequences, and (viii) a 739 nt E1 gene dataset (E1) containing 640 sequences. A 672 nt window was used for analyses of RdRp gene, as some of the additional sequences did not contain the entire gene region.

**Figure 5:**
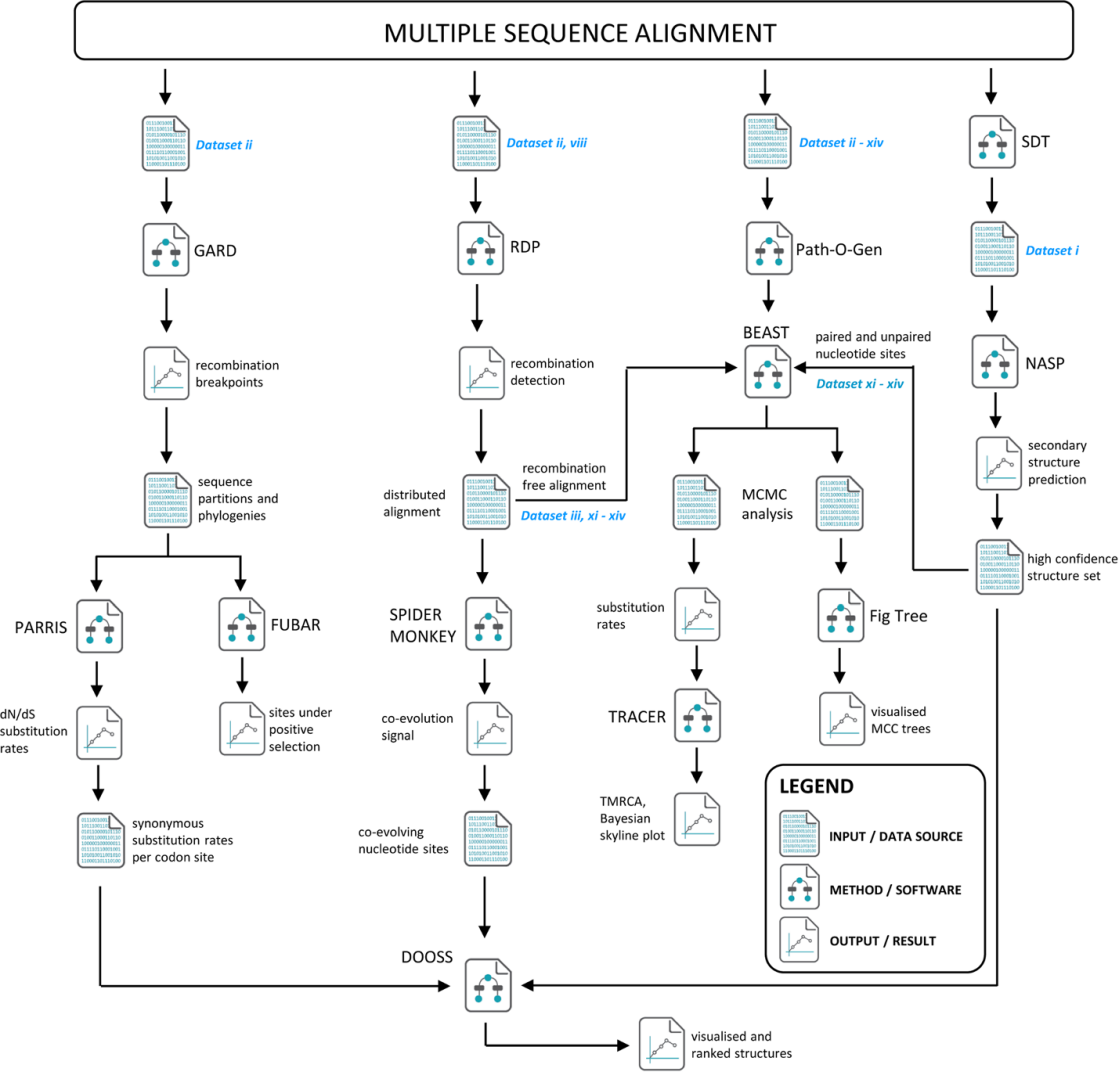
**Graphical representation of the analysis pipeline.** Sequence alignments were prepared using the MEGA package. The NASP method provides coordinates of potentially paired sites across the genome, using the representative sample of 10 full genome sequences as its input (dataset i, see Methods section). The GARD and RDP methods search for possible recombination breakpoints across the full alignment space and produce recombinant free partitions along with their corresponding phylogenies, which served as input for the PARRIS, FUBAR, and SPIDERMONKEY methods. Both PARRIS and FUBAR were used to determine substitution rates across the coding regions, whereas SPIDERMONKEY was used to detect sites which may be coevolving while still mainting complementary base-pairings. The DOOSS program was used to annotate and rank the NASP predicted nucleic acid secondary structures using the data sources obtained from the selection analysis above (FUBAR and SPIDERMONKEY). BEAST analysis was carried out using datasets in which the potential recombinants detected by RDP were removed, in addition to removing nucleotide sites which were predicted by NASP to form part of the high confidence structure set (HCSS). TRACER was used to analyse the resulting trace files, whereas the BEAST generated MCC tree files were summarised and annotated using FigTree.

To test the effect of nucleic acid secondary structure and temporal biases on our substitution rate estimates, we created (ix) a filtered E1 dataset containing only the 34 E1 encoding sequence regions excised from the full genome dataset (x) a temporally balanced E1 dataset containing 45 sequences. To generate the temporally balanced E1 dataset, we sorted the E1 dataset sequences into their respective decades and a maximum of 13 sequences from each decade were randomly selected for analysis, as this was the number of sequences available from the 1960s. For the 1970s and 1980s that contained less than 13 sequences, all the sequences were used in each replicate run. This process was repeated to generate 10 replicate datasets, each of which was analysed independently. (xi) an E1 recombination-free dataset of 638 sequences with all sites removed that were predicted to be base-paired within nucleic acid secondary structures identified by the computer program NASP (E1 rec.free UnPR; see below for method details; [19]), (xii) an E1 recombination-free dataset of 638 sequences containing only sites that were predicted by NASP to be base-paired (E1 rec.free PR) (xiii) a full genome recombination-free dataset of 32 sequences with all sites removed that were predicted to be base-paired within nucleic acid secondary structures (Full Genome rec.free UnPR) and (xiv) a full genome recombination-free dataset of 32 sequences containing only sites that were predicted by NASP to be base-paired (Full Genome rec.free PR). See Figure 5 for a graphical representation of the relationship between these datasets, as well as an analysis pipeline and rational behind the software used during this study.

### Evolutionary model selection

The best-fit nucleotide substitution model was estimated using MEGAv5.05 [39], and the degree of clock-like evolution was evaluated using root-to-tip genetic distance vs. sampling date regression analyses as implemented in the computer program, Path-O-Gen v1.4 [35] (dataset ii – xiv). Identification of the best-fit combined molecular clock and demographic model was determined using Bayes factor tests calculated as the ratio of the marginal likelihoods of the alternative models as determined using the computer program Tracer v.1.5 [41].

### Identification of nucleic acid secondary structures within RV genomes

Computational identification of evolutionarily conserved RV genome-wide nucleic acid secondary structure was achieved using the computer program NASP with default settings [19]. NASP uses the computer program hybrid-ss [18], to predict ensembles of plausible secondary structural elements evident within the genomes of ten RV genome sequences that reflect a representative sample of global RV genotype diversity (dataset i; see Additional file 4). These structural elements were ranked according to both their sizes, and their degrees of evolutionary conservation. NASP then used a series of nucleotide-shuffling permutation tests to determine which of the structures in this ranked list (known as the HCSS) represent RV genomes containing predicted folds with lower associated minimum free energies (MFE) than could be accounted for by chance.

Individual structural elements predicted by NASP were visualised and ranked in order of their likely biological functionality using DOOSS v1.0 [42]. Ranking was done according to the individual structure’s: (i) associated degrees of conservation (determined by NASP); (ii) degrees of synonymous substitution rate reduction at codon sites containing paired nucleotides (determined by PARRIS); (iii) the amount of evidence of complementary coevolution between nucleotides predicted to be base-paired, as determined by a SPIDERMONKEY-based method described in [26]; see Additional file 2.

Synonymous substitution rates at codon sites within the coding regions were estimated using the maximum likelihood phylogenetic-based selection characterization methods PARRIS [24] and FUBAR [23]. To determine the probabilities that individual nucleotides predicted to be paired (NASP-yielded HCSS) were coevolving in a way consistent with selection favouring the maintenance of base-pairing, we used a modification of the SPIDERMONKEY method [25].

We also tested for evidence of genome-wide associations between (i) base-pairing within the HCSS at codon sites and decreased synonymous substitution rates and (ii) base-pairing in the HCSS and sites detectably coevolving in a complementary fashion. The first of these tests compared the median synonymous substitution rates (determined by PARRIS) estimated at third codon positions between paired and unpaired sites using a Mann Whitney U-test. The second employed a Fishers exact test for an association between complementarily coevolution between site pairs (site pairs classified as complementarily coevolving or not by the SPIDERMONKEY-based method) and base-pairing between site pairs (site pairs classified as being base-paired or not by NASP).

### Recombination detection

Recombination can have a pronounced undesirable effect on the accurate inference of phylogenetic trees [27, 28], the estimation of precise nucleotide substitution rates [43] and the inference of positive selection [44]. To account for the potentially confounding effects of recombination within our RV datasets, we first analysed the 34 full-genome RV sequence dataset for evidence of inter and intra-strain recombination using RDP4.17 [33]. Using this program we were able to characterise probable recombination events, identify recombinants and likely parental sequences, and localize possible recombination breakpoints. Only potential recombination events detected by three or more out of the seven independent recombination detection methods implemented in RDP4.17 were considered as genuine recombination events. The Genetic Algorithm for Recombination Detection (GARD) [45] was also used to detect recombination breakpoints.

### Positive selection analyses

Because positive selection results in the fixation of advantageous mutations at a faster rate than neutral mutations, it can have a pronounced undesirable effect on the accurate estimation of precise long-term nucleotide substitution rates. To test whether there is evidence for positive selection acting at codon positions within the RV genome, we analysed the full genome dataset (dataset ii) using the fixed effects likelihood-based parametric selection inference method, FUBAR [23] implemented on the DATAMONKEY web server [46, 47].

### Bayesian phylogenetic analyses

A Bayesian Markov chain Monte Carlo (MCMC) method implemented in BEAST v.1.8.0 [48] was used to estimate evolutionary rates and times to the most recent common ancestral (TMRCA) sequences for all of the RV datasets described in Additional file 4. Four different evolutionary model combinations were investigated including either the non-parametric (Bayesian skyline plot) or parametric (constant population size) demographic models together with either strict or uncorrelated lognormal relaxed molecular clock models. For each dataset, between three and ten independent replicate runs were performed, ranging between 2.0 × 10^6^ and 4.0 × 10^8^ steps in length in the Markov chain using BEAST. As mentioned above Bayes factor tests were employed to identify the best-fit evolutionary models. All analyses were continued until the effective sample sizes (ESS) of all relevant model parameters were above 200: A criterion that ensured ample mixing of the Markov chain and parameter sampling prior to convergence of the MCMC chains. Similar results from independent runs of the Markov chains were combined using the program LogCombiner v1.8.0, which is also available in the BEAST package [48].

## Electronic supplementary material

Additional file 1: Figure S1: Rubella virus genome organization. A schematic representation of the monopartite, linear rubella virus genome. The genome contains a 5’-methylated nucleotide cap and a 3’-polyadenylated tail. The two open reading frames encoding the non-structural- (P150, P90) and structural polyproteins (CP, E2, E1), are represented by 2 distinct boxes, and the untranslated regions (UTR) as lines. Gene boundaries within the coding regions are indicated by solid vertical lines. The genomic RNA serves as mRNA for the translation of the non-structural proteins, or as a template for anti-sense genomic RNA synthesis. The non-structural proteins in turn, encode the viral proteins responsible for genome replication, by utilizing the cellular translational machinery. Embedded within the P150 gene are the methyl transferase and protease domains. Domains encoding the helicase and RNA-dependent RNA polymerase (RdRp) are located within the P90 gene. Gene regions are drawn to scale with respect to isolate [GenBank:JN635281]. (PNG 64 KB)

Additional file 2: Table S1: Consensus ranking of secondary structures in the high-confidence structure set is based on base-pairing conservation score, associated synonymous substitution rate and degree of co-evolution. Previously well-characterized structures are highlighted in yellow, while the top fifteen ranked structures are highlighted in green (see also Figure 1). (XLSX 19 KB)

Additional file 3: Figure S2: Example of nucleotide secondary structure of rubella virus (RV). This structure (labelled SL2) has been previously proposed [20] to interact with human calreticulin (CAL). The rank ratio shows the consensus rank of the structure over the total number of structures predicted to form part of the high-confidence structure set (see Figure 1 and Additional file 2). Site-to-site variations in synonymous substitution rates are reflected by colours ranging from blue to green (see colour key). Nucleotides falling outside the coding region are shaded in grey. The proposed CAL binding site (U-U bulge), is highlighted in orange, while the stem-loop region critical for RV-CAL interaction and the stop codon are highlighted in purple and red, respectively. (PNG 207 KB)

Additional file 4: Table S2: A full description of the rubella virus sequences and datasets used in this study, including the accession number, genotype assignment, collection date, country of origin and dataset assignment. (XLSX 56 KB)

Additional file 5: Figure S3: Estimates of the mean date and 95% HPD’s of the time to the most recent common ancestor (TMRCA) for the different RV sequence datasets under a constant population size and relaxed-clock model. (TIFF 243 KB)

## Abbreviations

RV: Rubella virus
CRS: Congenital rubella syndrome
WHO: World Health Organization
ORF: Open reading frame
NSP: Non-structural proteins
SP: Structural proteins
HCSS: High confidence structure set
TMRCA: Time to the most recent common ancestor
MCC: Maximum clade credibility
HPD: Highest Posterior Density
MFE: Minimum free energies
MCMC: Markov chain Monte Carlo
ESS: Effective sample sizes.
