# Supplementary figures and images for: The influence of secondary structure, selection and recombination on rubella virus nucleotide substitution rate estimates
Source: Virol J. 2014 Sep 16;11:166. doi: 10.1186/1743-422X-11-166 (PMC4175276; doi:10.1186/1743-422X-11-166)

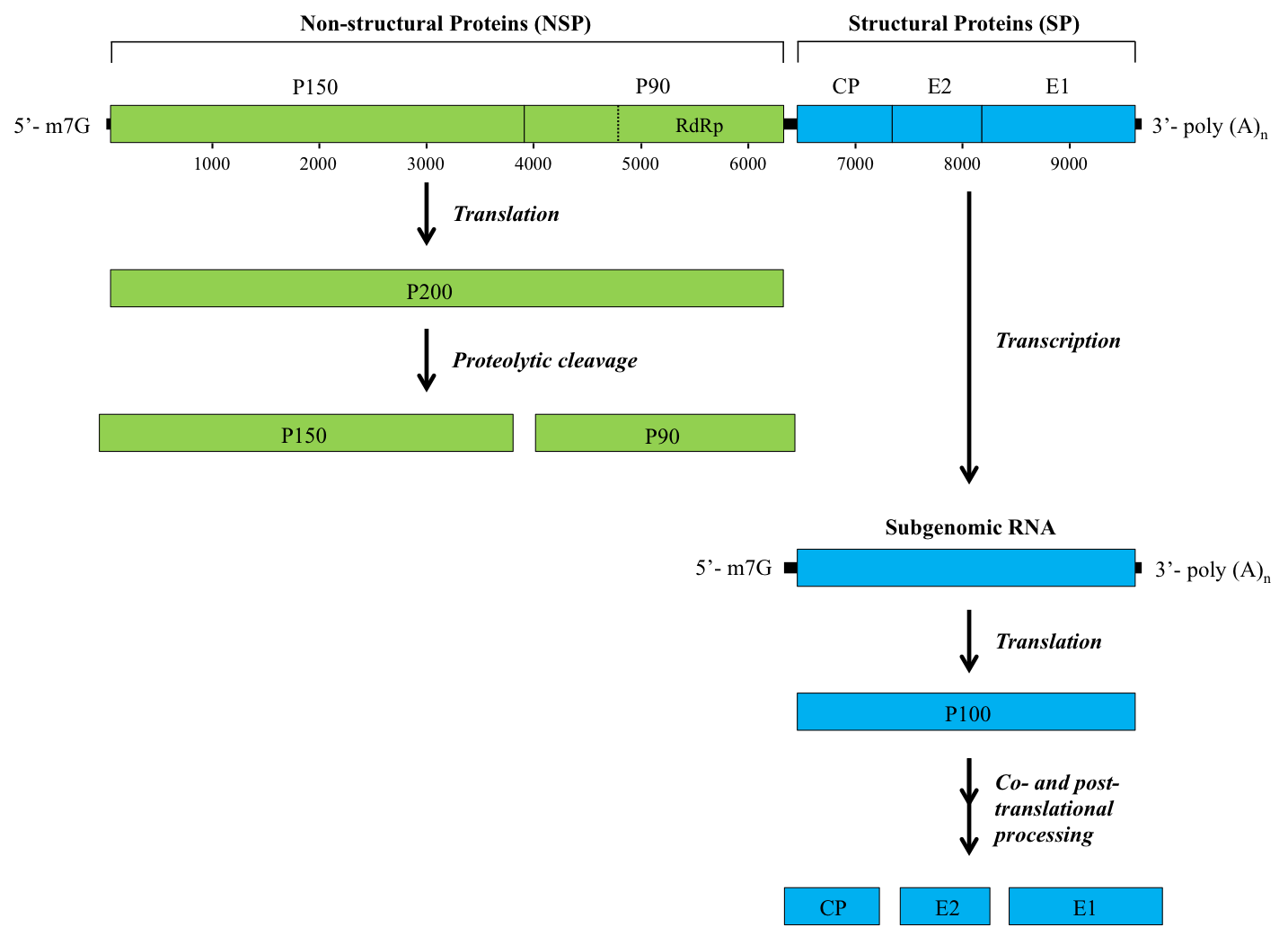

Supplement: Supplementary file 1 — Additional file 1: Figure S1: Rubella virus genome organization. A schematic representation of the monopartite, linear rubella virus genome. The genome contains a 5’-methylated nucleotide cap and a 3’-polyadenylated tail. The two open reading frames encoding the non-structural- (P150, P90) and structural polyproteins (CP, E2, E1), are represented by 2 distinct boxes, and the untranslated regions (UTR) as lines. Gene boundaries within the coding regions are indicated by solid vertical lines. The genomic RNA serves as mRNA for the translation of the non-structural proteins, or as a template for anti-sense genomic RNA synthesis. The non-structural proteins in turn, encode the viral proteins responsible for genome replication, by utilizing the cellular translational machinery. Embedded within the P150 gene are the methyl transferase and protease domains. Domains encoding the helicase and RNA-dependent RNA polymerase (RdRp) are located within the P90 gene. Gene regions are drawn to scale with respect to isolate [GenBank:JN635281]. (PNG 64 KB) [file 12985_2014_2492_MOESM1_ESM.png]

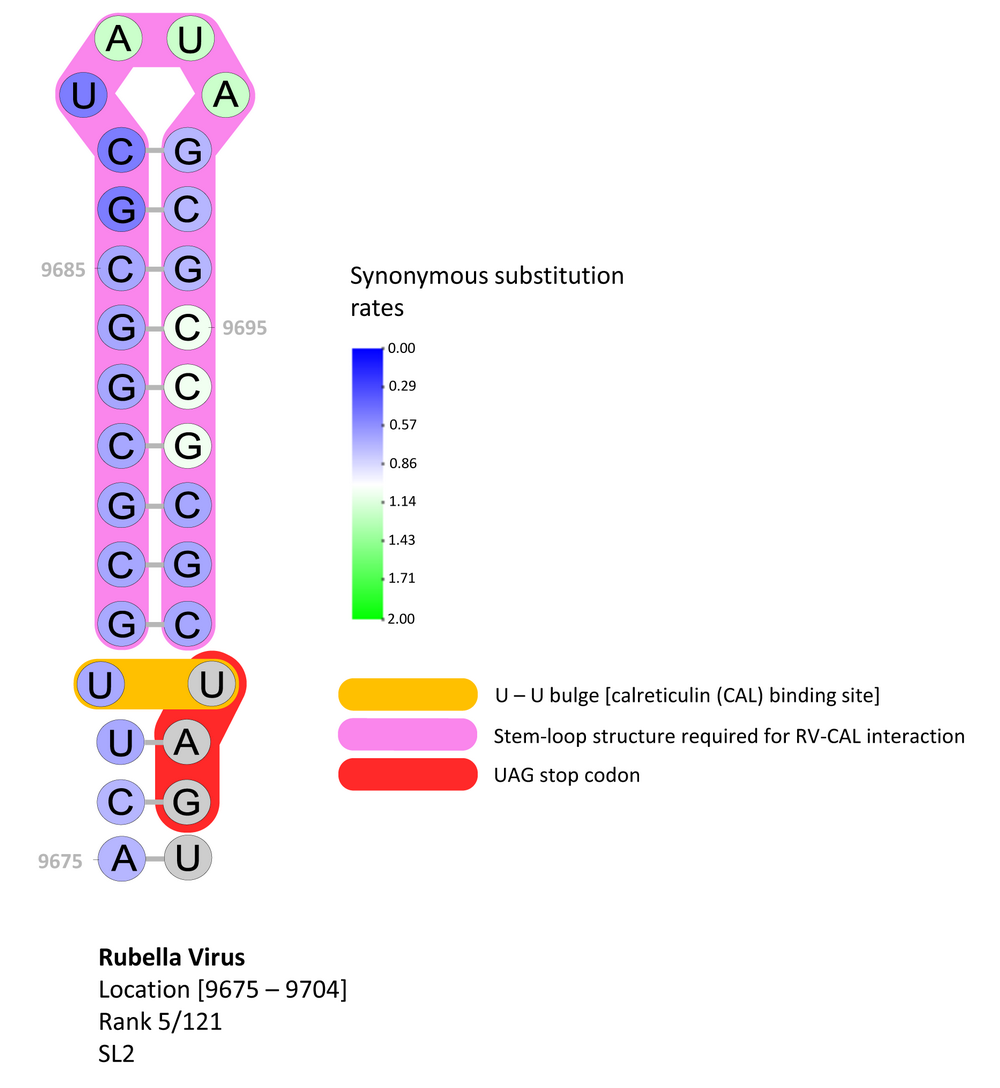

Supplement: Supplementary file 3 — Additional file 3: Figure S2: Example of nucleotide secondary structure of rubella virus (RV). This structure (labelled SL2) has been previously proposed [20] to interact with human calreticulin (CAL). The rank ratio shows the consensus rank of the structure over the total number of structures predicted to form part of the high-confidence structure set (see Figure 1 and Additional file 2). Site-to-site variations in synonymous substitution rates are reflected by colours ranging from blue to green (see colour key). Nucleotides falling outside the coding region are shaded in grey. The proposed CAL binding site (U-U bulge), is highlighted in orange, while the stem-loop region critical for RV-CAL interaction and the stop codon are highlighted in purple and red, respectively. (PNG 207 KB) [file 12985_2014_2492_MOESM3_ESM.png]

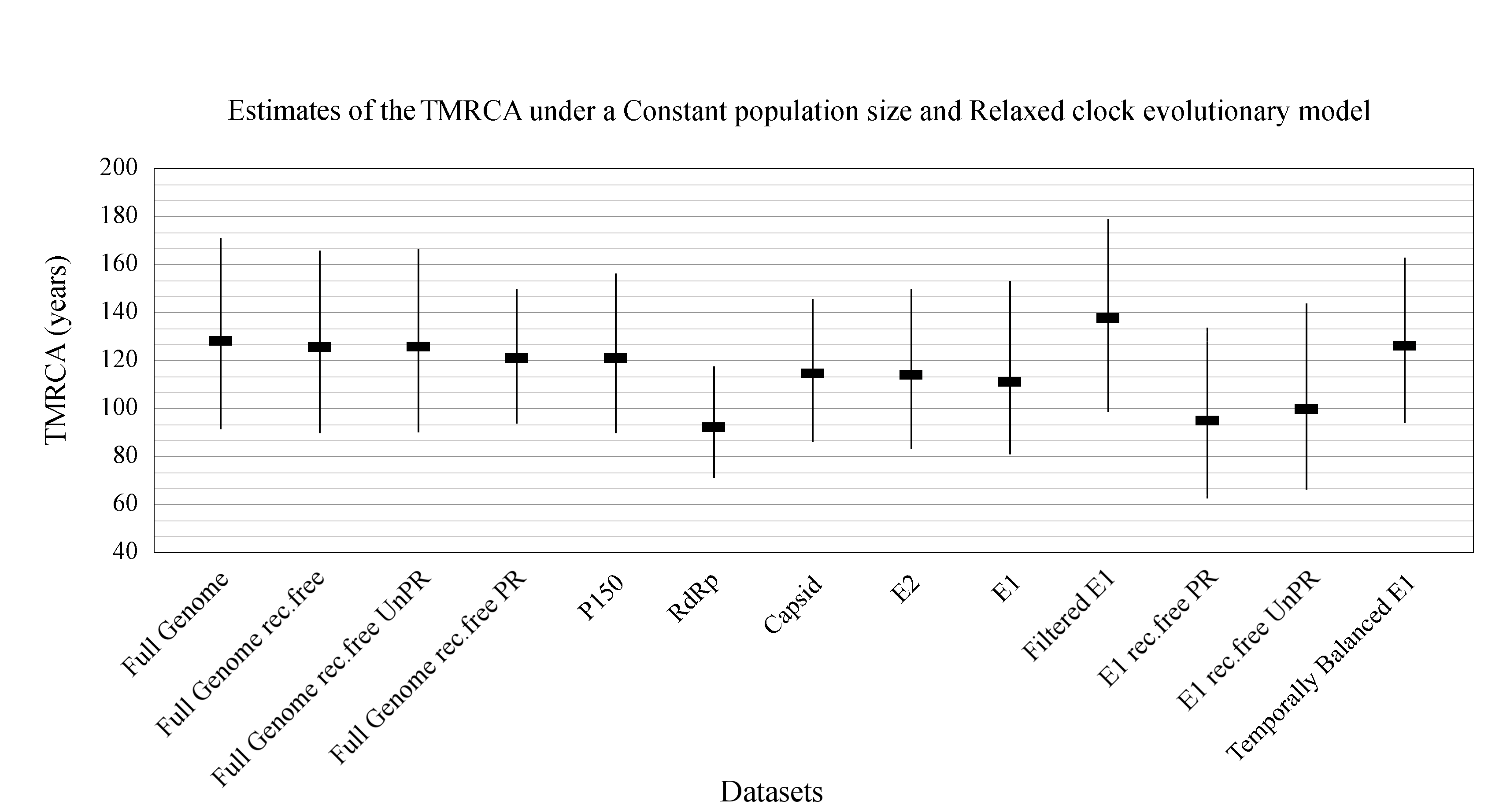

Supplement: Supplementary file 5 — Additional file 5: Figure S3: Estimates of the mean date and 95% HPD’s of the time to the most recent common ancestor (TMRCA) for the different RV sequence datasets under a constant population size and relaxed-clock model. (TIFF 243 KB) [file 12985_2014_2492_MOESM5_ESM.tiff]
